# Microangiopatía trombótica (MAT) asociada al embarazo: papel del laboratorio clínico en el diagnóstico diferencial

**DOI:** 10.1515/almed-2023-0138

**Published:** 2024-03-22

**Authors:** Patricia Ramos Mayordomo, Marta Capilla Díez, Danay Areli Ticona Espinoza, María Verónica Torres Jaramillo, Nathalie Martínez Tejeda, Thalia Gloria Ticona Espinoza, Cristina Colmenero Calleja, Virginia Fraile Gutiérrez

**Affiliations:** Servicio de Análisis Clínicos, 16918 Hospital Universitario Río Hortega, Valladolid, Castilla y León, España; Servicio de Nefrología, 16918 Hospital Universitario Río Hortega, Valladolid, Castilla y León, España; Servicio de Medicina Intensiva, 16918 Hospital Universitario Río Hortega, Valladolid, Castilla y León, España

**Keywords:** anemia hemolítica microangiopática, eculizumab, microangiopatía trombótica, preeclampsia, síndrome de HELLP, síndrome hemolítico urémico atípico

## Abstract

**Objetivos:**

La microangiopatía trombótica (MAT) se define por trombocitopenia, anemia hemolítica microangiopática y daño de órganos diana. El embarazo está asociado con varias formas de MAT como preeclampsia (PE), síndrome de HELLP, púrpura trombótica trombocitopénica (PTT) y síndrome hemolítico urémico (SHU). Cuando SHU se produce por desregulación de la vía alternativa del complemento se denomina SHU atípico (SHUa). El diagnóstico diferencial es complejo, ya que comparten características clínicas, siendo importante realizarlo precozmente para instaurar tratamiento específico y mejorar el pronóstico.

**Caso clínico:**

Primigesta de 43 años ingresa a la edad gestacional de 33 semanas, con diagnóstico de preeclampsia grave y sufrimiento fetal por lo que se realiza cesárea urgente. En el puerperio inmediato, presenta insuficiencia hepática aguda y fracaso renal anúrico en contexto de síndrome de HELLP, anemia, trombopenia, hipertensión arterial (HTA) y alteraciones neurológicas. Se realiza estudio de MAT y diagnóstico diferencial evidenciando además SHUa asociado al embarazo. Se inicia tratamiento con Eculizumab presentando buena respuesta y progresiva mejoría clínica y analítica.

**Conclusiones:**

El SHUa es una enfermedad rara y multifactorial con elevada mortalidad antes de la aparición del Eculizumab. Debido al complejo diagnóstico, el laboratorio clínico tiene un papel clave en el diagnóstico diferencial y abordaje.

## Introducción

Las microangiopatías trombóticas (MAT) que se presentan en el embarazo o puerperio pueden ser difíciles de diagnosticar y tener impacto en la madre y en el feto. Existen diferentes formas de MAT asociadas al embarazo, como preeclampsia (PE), síndrome de HELLP, púrpura trombótica trombocitopénica (PTT), síndrome hemolítico urémico (SHU), hígado graso agudo del embarazo, síndrome antifosfolípido e incluso hemorragias post-parto graves. Estas patologías comparten síntomas y signos por lo que es importante realizar un correcto diagnóstico diferencial [1, 2].

La PE se caracteriza por lesión endotelial previa a las manifestaciones clínicas, multisistémica y progresiva. Complica alrededor del 3 % de las gestaciones y es causa importante de morbimortalidad materna y perinatal [3]. Mientras que el síndrome HELLP es una complicación obstétrica caracterizada por hemólisis, elevación de enzimas hepáticas y trombocitopenia [4, 5]. Es una variante o complicación de PE grave, pero algunos autores consideran que es un síndrome no relacionado con ella porque en un 15–20 % de los casos se presenta sin proteinuria ni HTA [4].

El SHU se define por la tríada anemia hemolítica microangiopática no inmune, trombocitopenia e insuficiencia renal aguda (IRA). Las lesiones histológicas se caracterizan por la aparición de MAT sistémica, que afecta preferentemente a los vasos intrarrenales. La mayoría de los casos son causados por infección entérica por *Escherichia coli* productora de toxina Shiga (STEC), dando lugar a SHU típico. En aproximadamente 10 % de los casos se produce debido a desregulación de la vía alternativa del sistema del complemento que provoca daño endotelial y fenómenos de MAT sistémica. Este tipo de SHU se denomina SHU atípico (SHUa) y es una enfermedad rara [1, 6].

Las pruebas de laboratorio nos permiten realizar un adecuado diagnóstico diferencial con el fin de establecer un diagnóstico precoz para instaurar tratamiento temprano, disminuyendo la morbimortalidad. Es importante destacar la necesidad del abordaje multidisciplinar para mejorar la atención sanitaria a estos pacientes.

## Caso clínico

Paciente primigesta de 43 años, monorrena por reflujo ureterovesical en la infancia con función renal normal previa, diagnosticada de PE precoz en semana 25 de gestación. Presentaba los siguientes marcadores angiogénicos: forma soluble de tirosina quinasa-1 similar al fms (sFlt-1): 13.901 pg/mL (24–28 semanas: 618–3.205 pg/mL), factor de crecimiento placentario (PlGF): 56,22 pg/mL (24–28 semanas: 130–1.108 pg/mL), ratio sFlt-1/PlGF: 247,26 (20–34 semanas: >85 es indicativo de sospecha de PE precoz).

Ingresa en otro centro hospitalario a edad gestacional de 33 semanas, con diagnóstico de retraso del crecimiento intrauterino y PE grave. Se objetiva sufrimiento fetal con bradicardia mantenida, por lo que se realiza cesárea urgente. Previamente, presentaba creatinina: 1,09 mg/dL (0,51–0,95 mg/dL), alanina aminotransferasa (ALT): 30 U/L (10–49 U/L), hemoglobina: 12,1 g/dL (12–15 g/dL) y plaquetas: 328×10^9^/L (140–450×10^9^/L).

Tras la cirugía presenta tensiones arteriales elevadas y se evidencia deterioro de función renal, fallo hepático grave y alteración de parámetros hematológicos. Aumenta el deterioro clínico y analítico con creatinina: 1,79 mg/dL (0,51–0,95 mg/dL), ALT: 266 U/L (10–49 U/L), AST: 621 U/L (≤34 U/L), lactato deshidrogenasa (LDH): 1.201 U/L (120–246 U/L), procalcitonina: 64,5 ng/mL (≤0,5 ng/mL), lactato: 4,4 mmol/L (0,5–1,6 mmol/L), hemoglobina: 10,1 g/dL (12–15 g/dL), plaquetas: 39×10^9^/L (140–450×10^9^/L) y dímero D: 35.200 ng/mL (0–500 ng/mL). Ante cuadro de insuficiencia hepática aguda grave en contexto de síndrome de HELLP, se contacta con nuestro hospital (Unidad de Referencia en trasplante hepático) y se traslada a la paciente a la Unidad de Cuidados Intensivos (UCI).

Debido a sospecha de MAT asociada al embarazo, se realiza diagnóstico diferencial para establecer diagnóstico precoz, ya que existen enfermedades graves con elevada morbimortalidad que comparten características clínicas y analíticas (Tabla 1) [4, 7], [8], [9].

**Tabla 1:** Diagnóstico diferencial de preeclampsia (PE), síndrome de HELLP, hígado graso agudo del embarazo, púrpura trombótica trombocitopénica (PTT) y síndrome hemolítico urémico (SHU).

Signos y síntomas/pruebas de laboratorio	Preeclampsia (PE)	Síndrome de HELLP	Hígado graso agudo del embarazo	Púrpura trombótica trombocitopénica	Síndrome hemolítico urémico
Hipertensión	100 %	85 %	50 %	20–70 %	80–90 %
Proteinuria	100 %	85 %	30–50 %	Asociado a hematuria	80–90 %
Anemia hemolítica	No	50–100 % severa	Poco frecuente	100 % severa	100 % severa
Lactato deshidrogenasa	Variable	>600	Variable	>1.000	>1.000
Plaquetopenia	>15×10^9^/L	>20×10^9^/L	>50×10^9^/L	<20×10^9^/L	>20×10^9^/L
Transaminasas	–	++	++	+/−	+/−
Insuficiencia renal	+/−	20 %	90–100 %	30 %	100 %
Hipoglucemia	No	No	Presente severa	No	No
Coagulación intravascular diseminada	No	Raro	Frecuente	Raro	Raro
ADAMTS-13 <10 %	Ausente	Ausente	Ausente	Presente	Ausente
sFlT-1/PlGF	>85 (PE precoz)	>85	<38	<38	<38
	>110 (PE tardía)				

PE, preeclampsia; sFlT-1/PlGF, forma soluble de tirosina quinasa-1 similar al fms/factor de crecimiento placentario. Modificado a partir de: Arigita M, y col [4].

Se inicia estudio de hemólisis intravascular inmunitaria y MAT. El aumento de LDH: 4.625 U/L (100–190 U/L), haptoglobina indetectable: <6 mg/dL (36–195 mg/dL), aumento de bilirrubina total: 5,91 mg/dL (0,2–1,1 mg/dL) y presencia de esquistocitos: 2 %, confirman la hemólisis *in vivo* intravascular. Sin embargo, el Test de Coombs directo e indirecto negativos descartan anemia hemolítica autoinmune.

Se determinó PLASMIC Score, herramienta de predicción clínica útil que permite diferenciar entre PTT y otras MAT, en los centros hospitalarios que no disponen de determinación de ADAMTS-13 en un tiempo <24 horas y se realiza en un laboratorio externo. El resultado fue 4 (riesgo bajo: considerar diagnósticos alternativos) y la actividad de ADAMTS-13: 15 % descartando PTT, ya que criterio diagnóstico es <5–10 % [1, 10, 11]. También se evaluó STEC siendo negativo, descartando SHU típico.

**Figura 1:**
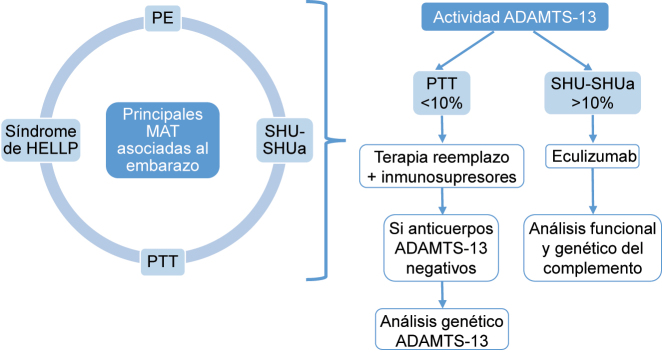
Resumen del criterio diagnóstico y tratamiento de elección según la actividad de ADAMTS-13. PE, preeclampsia; PTT, púrpura trombótica trombocitopénica; SHU, síndrome hemolítico urémico; SHUa, síndrome hemolítico urémico atípico.

Ante la mala evolución, necesidad de medidas de soporte desde ingreso en UCI (depuración extracorpórea combinada y plasmaféresis), IRA grave con anuria, ADAMTS-13 >5–10 % y tras excluir otras formas de MAT, la sospecha diagnóstica es SHUa asociado al embarazo [10]. Se solicita estudio funcional y genético del complemento para ver si existe desregulación con hiperactividad de la vía alternativa (valorando consumo de componentes o presencia de marcadores de activación) y se inicia tratamiento con Eculizumab el día +5 (Figura 1) [1, 11, 12]. Presentó progresivamente mejoría clínica y analítica con recuperación de la función renal y hepática, mejora hemodinámica, cese de la hemólisis *in vivo* y no precisando terapia de depuración extracorpórea ni plasmaféresis desde inicio con Eculizumab. Cabe destacar que los parámetros de las pruebas de función hepática no descendieron de manera progresiva debido a la complicación de un absceso hepático que se resolvió con drenaje y antibioterapia dirigida.

En la Tabla 2, podemos ver la evolución analítica desde su ingreso en nuestro hospital hasta el alta.

**Tabla 2:** Evolución analítica de la paciente a lo largo de su ingreso hospitalario en nuestro hospital.

Parámetro	Día												
	Post-cesárea	+1	+2	+3	+4	+5	+10	+15	+ 20	+25	+30	+35	+40
Potasio, mmol/L (VR: 3,5–5,1)	5,8	3,6	4	3,4	2,9	3,4	3,8	4,6	3,6	3,1	3,4	3,1	3,8
Urea, mg/dL (VR: 12,8–42,8)	86,2	32,6	24	31,7	49,2	39,8	68,6	106,7	174,3	61	38,1	38	21,4
Creatinina, mg/dL (VR: 0,6–1,1)	3,17	1,62	1,18	1,6	2,25	1,67	0,95	1,07	2,05	0,83	0,73	0,46	0,43
AST, U/L (VR: 0–35)	5.473		2.136	614	265	165	83	131	182	111	85		
ALT, U/L (VR: 1–35)	3.925	1.344	1.509	590	281	149	53	108	104	46	28	25	
GGT, U/L (VR: 0–38)	114		116	116	112	78	90	468	852	957	796		
Bilirrubina total, mg/dL (VR: 0,2–1,1)	5,91	6,87	8,45	9,74	12,45	14,53	18,28	14,06	18,28	16,15	13,13	13,04	
Bilirrubina directa, mg/dL (VR: 0–0,2)	2,39	2,81	3,28	3,75	6,02	7,16	12,55	8	12,18	8,79	8,06	7,76	
Bilirrubina indirecta calculada, mg/dL (VR: 0–1)	3,52	4,06	5,17	5,99	6,43	7,37	5,73	6,06	6,1	7,36	5,07	5,28	
FA, U/L (VR: 30–120)	471		449	350	538	318	117	443	682	842	875		
LDH, U/L (VR: 100–190)	4.625	5.130		4.680	2.630	1.050	662	366		367	362		
Procalcitonina, ng/mL (VR: 0–0,5)	138,41		49,98	35,69	38,67	39,69	5,43	2,92	1,81	1,06	0,82	0,46	
Amonio, µmol/L (VR: 18–72)	184	111	290	79	105	78	100	41	70	56	44	8	
Hb, g/dL (VR: 11,4–15,1)	7,2	8,6	9,2	9	8,7	9,8	9,1	8,8	8,7	8,4	7,7	8	8
Hb libre, g/dL (VR: 0–0,1)		0,4		0,5	1,2	0,2	0,8	0,2	0,2	0,2	0,2		
Plaquetas, ×10^9^/L (VR: 150–350×10^9^/L)	27	50	63	69	47	10	25	67	216	186	167	260	374
Dímero D, ng/mL (VR: 0–500)	32.907			19.836									
Fibrinógeno, mg/dL (VR: 180–420)	221	230	224	195	185	389	171	310	453	515	441	443	

VR, valores de referencia; AST, aspartato aminotransferasa; ALT, alanina aminotrasferasa; GGT, gamma-glutamil transferasa; FA, fosfatasa alcalina; LDH, lactato deshidrogenasa; Hb, hemoglobina.

Los resultados del estudio del complemento fueron: C3: 34 mg/dL (70–150 mg/dL), C4: 2 mg/dL (14–60 mg/dL), CH50: <13 U/mL (42–95 U/mL), FH: 77 μg/mL (90–285 μg/mL), FI: 18 μg/mL (20–40 μg/mL), anticuerpos anti-FH: negativos y MCP: 90 % (77–121 %). Se evidencian niveles particularmente bajos de C3 y C4, proteínas del complemento de síntesis hepática, lo que se justifica con el fallo hepático. Por tanto, no hay presencia de niveles de C3dg sugestivos de activación de C3.

Además, tampoco se identifican alteraciones genéticas en panel NGS 14 genes del complemento (*CFH*, *CFHR1*, *CFHR2*, *CFHR3*, *CFHR4*, *CFHR5*, *C3*, *CFI*, *MCP (CD46)*, *CFB*, *THBD*, *DGKE*, *CFP*, *ADAMTS13*), la mayoría relacionados con SHUa [13].

A la vista de los resultados, la paciente presentó PE grave y síndrome de HELLP con fallo hepático hiperagudo en el puerperio inmediato. Ante la mala evolución con persistencia de anemia hemolítica e IRA con anuria y tras realizar diagnóstico diferencial, se sospecha que además padece SHUa asociado al embarazo. Sin embargo, no se identifican alteraciones genéticas predisponentes y existe disminución de las proteínas del complemento con síntesis hepática debido al fallo hepático. Pero, el curso clínico y la respuesta al tratamiento con Eculizumab son indicativos de SHUa secundario asociado al embarazo. Esto sugiere que existen desencadenantes de la hiperactividad del complemento, como el fenotipo proinflamatorio y procoagulante activado de las células endoteliales vasculares, que son capaces de producir el mismo cuadro clínico que en un SHUa primario causado por alteraciones genéticas [14].

## Discusión

Las MAT durante el embarazo y el puerperio son síndromes de diagnóstico complejo con entidades de expresión clínica variable, pudiendo solaparse entre ellas. Debido a esta complejidad, el laboratorio clínico tiene un papel clave en el diagnóstico diferencial y en su abordaje. La identificación del SHUa asociado al embarazo es diagnóstico de exclusión cuando se han descartado otras causas de MAT. El diagnóstico tardío y la no instauración de tratamiento temprano pueden poner en peligro la vida.

El SHUa es una enfermedad rara, compleja y multifactorial caracterizada por anemia hemolítica microangiopática (hemoglobina <10 mg/dL, test de Coombs directo negativo, LDH elevada, descenso de haptoglobina, reticulocitosis, presencia de esquistocitos), trombocitopenia (plaquetas <150×10^9^/L o descenso >25 % desde inicio) e IRA [1, 2, 7, 8].

Debido a la hemólisis *in vivo* que presenta la paciente, las concentraciones de ALT, AST, LDH y potasio son propias de la situación clínica y no debido a hemólisis *in vitro* ocasionante de interferencia. Por lo tanto, se considera mala praxis no informarlas. Sin embargo, existe una interferencia analítica metodológica que infraestima la concentración de bilirrubina, causada por esta hemólisis.

**Figura 2:**
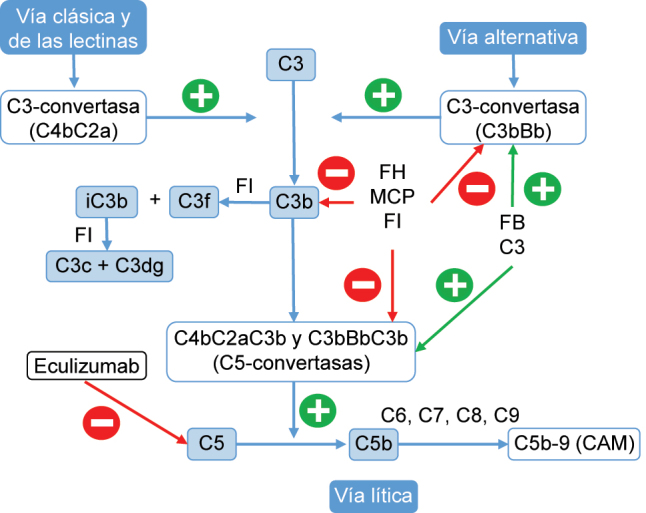
Vías de activación del sistema del complemento y punto de acción de eculizumab en el síndrome hemolítico urémico atípico (SHUa). La activación del complemento por la vía alternativa, la vía clásica o la vía de las lectinas ocasiona deposición de grandes cantidades de C3b sobre la membrana celular del activador, lo que conduce a su opsonización y a la activación del C5 (vía lítica), que conduce a la formación del complejo de ataque a la membrana (CAM) y la lisis celular. CAM, complejo de ataque a la membrana; FI, factor I; FH, factor H; FB, factor B; MCP, proteína cofactor de membrana.

El tratamiento del SHUa se basa en el uso de Eculizumab, anticuerpo IgG2/4κ monoclonal humanizado que se une a la proteína del complemento C5, bloqueando su escisión e impidiendo la generación del complejo C5b-9 [15]. La desregulación de la vía alternativa del complemento conlleva una activación incontrolada de C5 que provoca daño en estructuras propias mediante la formación del complejo de ataque de membrana. El bloqueo de la vía terminal reduce rápida y sostenidamente este proceso (Figura 2) [1, 2, 12, 15].

El bloqueo del complemento con Eculizumab se asocia con una buena respuesta clínica y una reversibilidad de la MAT, reduciendo significativamente los biomarcadores de inflamación, riesgo trombótico, daño endotelial y orgánico. Esto sugiere que la desregulación del complemento sin presencia de alteraciones genéticas tiene probablemente un papel importante en el SHUa, predisponiendo a su desarrollo ya que aproximadamente en un 40 % de pacientes no se identifica susceptibilidad genética o anticuerpos anti-FH [1, 6, 11].

La clasificación de las MAT es un tema de actualidad y existe debate en la comunidad médica debido al constante avance en el conocimiento de la fisiopatología. Además, la importancia del abordaje multidisciplinar de enfermedades raras como SHUa, es esencial para establecer un diagnóstico precoz y mejorar el pronóstico.

## Puntos de aprendizaje

– El diagnóstico de MAT asociada al embarazo es complejo, por lo que el laboratorio clínico tiene un papel clave en el diagnóstico diferencial y abordaje debido a la importancia de las interconsultas realizadas al laboratorio por los clínicos.
– El tratamiento temprano de SHUa con Eculizumab ha demostrado mejoría clínica en el pronóstico.
– El riesgo de SHUa en embarazos posteriores es aproximadamente 25 %, siendo menor si no se identifica ninguna alteración genética del complemento.
– La identificación de la hemólisis *in vivo* debido a la situación clínica y la interferencia analítica metodológica causada por esta hemólisis es fundamental, por lo que los especialistas de medicina de laboratorio añaden valor esencial a la fase postanalítica en la emisión de los informes de resultados.
– Es necesario un abordaje multidisciplinar para mejorar la atención sanitaria de los pacientes, sobre todo en enfermedades raras, porque muestra beneficios clínicos, sociales y económicos.

## References

[1] Campistol JM, Arias M, Ariceta G, Blasco M, Espinosa L, Espinosa M (2015). Documento de consenso: actualización en síndrome hemolítico urémico atípico: diagnóstico y tratamiento. Nefrología.

[2] Fakhouri F, Scully M, Provot F, Blasco M, Coppo P, Noris M (2020). Management of thrombotic microangiopathy in pregnancy and postpartum: report from an international working group, The American Society of Hematology. Blood.

[3] Romero MA, Orós D, Vigil-De Gracia P, Fabre E (2018). Estados hipertensivos del embarazo.

[4] Arigita M, Martínez GS (2020). Síndrome HELLP: controversias y pronóstico. Hipertens Riesgo Vasc.

[5] Sociedad Española de Ginecología y Obstetricia (2020). Guía de Asistencia Práctica: trastornos hipertensivos en la gestación. Prog Obstet Ginecol.

[6] Bruel A, Kavanagh D, Noris M, Delmas Y, Wong E, Bresin E (2017). Hemolytic uremic syndrome in pregnancy and postpartum. Clin J Am Soc Nephrol.

[7] Chinchilla KA, Vijayan M, Taveras B, Jim B (2020). Complement-mediated disorders in pregnancy. Adv Chronic Kidney Dis.

[8] Scully M (2021). How to evaluate and treat the spectrum of TMA syndromes in pregnancy. Hematology Am Soc Hematol Educ Program.

[9] Gaggl M, Aigner C, Csuka D, Szilágyi A, Prohászka Z, Kain R (2018). Maternal and fetal outcomes of pregnancies in women with atypical hemolytic uremic syndrome. J Am Soc Nephrol.

[10] Contreras E, de la Rubia J, del Río-Garma J, Díaz-Ricart M, García-Gala JM, Lozano M (2015). Guía diagnóstica y terapéutica de las microangiopatías trombóticas del Grupo Español de Aféresis. Med Clin.

[11] Fakhouri F, Frémeaux-Bacchi V (2021). Thrombotic microangiopathy in aHUS and beyond: clinical clues from complement genetics. Nat Rev Nephrol.

[12] Scully M, Neave L (2023). Etiology and outcomes: thrombotic microangiopathies in pregnancy. Res Pract Thromb Haemost.

[13] Fakhouri F, Roumenina L, Provot F, Salle M, Caillard S, Couzi L (2010). Pregnancy-associated hemolytic uremic syndrome revisited in the era of complement gene mutations. J Am Soc Nephrol.

[14] Praga M, Rodríguez de Córdoba S (2019). Secondary atypical hemolytic uremic syndromes in the era of complement blockade. Kidney Int.

[15] Agencia Española de Medicamentos y Productos Sanitarios (2014). Informe de Posicionamiento Terapéutico PT/V1/19112014: Informe de Posicionamiento Terapéutico de Eculizumab (Soliris^®^) en el Síndrome Hemolítico Urémico atípico.

